# Document review of the paper-based implementation of the Framework and strategy for disability and rehabilitation in Gauteng, South Africa

**DOI:** 10.1371/journal.pone.0315778

**Published:** 2025-02-25

**Authors:** Naeema Ahmad Ramadan Hussein El Kout, Natalie Benjamin-Damons, Sonti Imogene Pilusa

**Affiliations:** Department of Physiotherapy, School of Therapeutic Sciences, Faculty of Health Sciences, The University of the Witwatersrand, Johannesburg, South Africa; Sefako Makgatho Health Sciences University, SOUTH AFRICA

## Abstract

**Background:**

The prevalence of disability is on the rise globally and in South Africa, with a high number of unmet needs and poor actualisation of the health rights of persons with disabilities. A tool to realise these rights is health policy, such as the framework and strategy for disability and rehabilitation (2015-2022)(FSDR). There are limited data on the implementation outcomes of the FSDR.

**Objective:**

To review the implementation of the FSDR according to the paper-based provincial reports.

**Methods:**

The study conducted a document review and utilised a concurrent mixed-method design, combining qualitative and quantitative data extracted from paper-based evaluation templates developed by the South African National Department of Health (NDoH). The qualitative analysis involved thematic coding using the RE-AIM framework to examine the FSDR’s implementation across eight provinces, while quantitative data, such as frequencies and percentages, provided supplementary insights.

**Results:**

The quantitative results revealed that 87% of the reports from provinces reported physical accessibility to the FSDR, and 62% of provinces received training on the implementation of the FSDR. Only two out of eight provinces have conducted monitoring and evaluation since implementing the FSDR in 2015. Qualitative findings revealed poor reach and adoption of the FSDR owing to a lack of implementation training for end users. The lack of indicators resulted in poor maintenance of the FSDR, as well as the lack of human resources and equipment which resulted in the reduced efficacy of the FSDR.

**Conclusion:**

The FSDR has not achieved its full level of implementation due to numerous barriers, such as lack of resources, human capacity, and training on implementation.

## Introduction

Globally, 16 percent of the world’s population lives with disabilities, with an expected exponential increase by 2050 [1]. Among this high global prevalence, 80% of global disability cases occur in developing countries [2]. The prevalence of disability in Africa ranges from 5 percent to 20% across different countries, and is attributed to poor access to health care, conflict and forced displacement [3,4]. In South Africa, the disaggregation of disability-related data has led to potentially unaccounted cases [5]. Despite this, it is estimated that the national disability prevalence is 7.5%, which is attributed to infectious diseases, non-communicable diseases, trauma, and delayed initiation of health interventions [6,7] The increase in disability has increased the need for rehabilitation [6,7,8].

South Africa has the Integrated National Disability Strategy (INDS) of 1997 which paved the way for the Framework and Strategy for Disability and Rehabilitation (FSDR) (2015–2022). The INDS plays a crucial role in shaping disability policy in South Africa, and any analysis must consider its impact and context. While the FSDR is a significant recent policy, the INDS provides foundational principles and strategies that continue to influence current policies [9]. The FSDR was developed by the National Department of Health (NDOH) in South Africa (SA) for 2015–2020 [10]. The FSDR aims to guide and improve access to rehabilitation services for persons with disabilities in South Africa [11]. With the COVID-19 pandemic in the early months of 2020, the FSDR will be extended until 2022. Upon the conclusion of the FSDR, the National Department of Health (NDOH) had been tasked with evaluating the implementation of the FSDR within each province of South Africa. Few studies have reviewed the FSDR. A study by colleagues [22] reviewed the FSDR by means of a policy analysis and revealed that there are numerous gaps in the development process of the FSDR, such as a lack of end-user engagement and reduced budget allowance for policy development. However, no study has evaluated the implementation of the FSDR in Gauteng Province at the ground level.

Disability extends beyond social and biological constructs of restricted functionality [13]. Disability is the outcome of the relationship between environmental and personal factors, and a health condition which subsequently affects the way an individual can participate in daily life [14,15]. In the global and South African contexts, the health needs and rights of persons with disabilities have not been upheld because of the lack of data availability, policy inclusion, and poor end-user engagement [16]. A mechanism to realise these rights is the adequate development and implementation of public health policies [17]. Global efforts were made by the United Nations to protect the rights of persons living with disabilities through the development of the United Nations Convention on the Rights of Persons with Disabilities, to which South Africa is a signatory [18,19]. This subsequently mandated South Africa to have appropriate disability policies in place, such as the Framework and Strategy for Disability and Rehabilitation (FSDR) [20].

The importance of reviewing policy is that it identifies gaps in the processes and provides recommendations for future policies. The FSDR policy analysis conducted by colleagues [12] recommended an evaluation focused on the implementation of the FSDR in the Gauteng Province. With the current rise in the prevalence of disability globally and in South Africa, there is an increasing realisation that the rights of persons with disabilities have not been actualised [20]. The right to access health care, such as rehabilitation services, can be actualised through the adequate implementation of a disability-related public health policy. The FSDR expired in 2022, and its implementation has not yet been evaluated [21]. This lack of evaluation can result in future disability policies that repeat previous mistakes and do not address gaps in service delivery and health outcomes [21].

## Methodology

### Study design

The study employed a document review methodology with a predominantly qualitative focus, supplemented by limited quantitative data. Thus a concurrent mixed-method design was used, where qualitative and quantitative data were extracted from documents for separate analysis and then integrated. The quantitative component was a cross-sectional descriptive study using descriptive statistics, while the qualitative component was a descriptive qualitative study that involved evaluating qualitative responses from paper-based implementation reports. This study is part of a bigger study [22].

The South African National Department of Health (NDOH) developed a paper-based evaluation template which was completed by rehabilitation managers in their respective provinces. The template explored the physical accessibility of the FSDR document, the extent of training on FSDR implementation within each province, and the level of monitoring and evaluation undertaken throughout the FSDR’s lifespan. The FSDR aims to define and guide rehabilitation service provision from a primary healthcare level through to a specialised hospital, to improve access to healthcare for persons with disabilities [21].

### Study setting

The study setting in this case refer to the geographic locations from which the data originated. These include the 8 provinces of South Africa that reported on the FSDR was implementation. This includes Gauteng, Mpumalanga, Northwest, Eastern Cape, Western Cape, Northern Cape, Kwa-Zulu Natal and Limpopo but data has been anonymysed by allocating provinces a number in the results section. The study did not directly engage with individuals but rather analysed reports collected by the National Department of Health (NDoH) from these provinces. The settings are described as the administrative and operational contexts of the provincial health departments where the FSDR implementation occurred.

### Data collection and analysis

The study employed a paper-based evaluation methodology, focusing on data collected from the nine provinces of South Africa through standardised templates developed by the National Department of Health (NDoH). These templates were designed to capture information on the accessibility, training, and monitoring and evaluation (M&E) of the Framework and Strategy for Disability and Rehabilitation (FSDR).

Each province completed the evaluation template, which was then submitted to the NDoH. The researcher obtained these completed reports from the NDoH rehabilitation directorate following formal permission granted via email. Once approved, the reports were sent to the researcher for analysis. Data access and analysis occurred between 7 September 2022 and 31 March 2023. The researcher played no role in the design of the templates or the data collection process but focused solely on analysing the reports provided by the NDoH. The study was conducted across the nine provinces, with data collected from their respective health departments.

The paper-based implementation provincial reports of the FSDR comprised questions on the accessibility of the FSDR in terms of electronic access, access to the FSDR on the NDOH website, and access to hard copies. The report also explored the level of training that was performed on the FSDR, as well as the level of monitoring and evaluation (M&E) that was performed within each province.

Quantitative data, though limited, were extracted as numerical references (e.g., frequencies and percentages). These data provided supplementary insights into specific aspects of the document’s implementation or outcomes.

#### Qualitative analysis.

Thematic analysis was conducted to identify recurring themes, subthemes, and patterns. The qualitative data were coded using MAXQDA version 2020.0 to ensure rigor and consistency. The data were analysed deductively using the RE-AIM framework. The deductive thematic analysis, guided by the RE-AIM framework, systematically coded and interpreted qualitative data using MAXQDA software. The researcher familiarised themselves with the data, coded it according to the framework’s categories—Reach, Effectiveness, Adoption, Implementation, and Maintenance—and developed initial codes into broader themes. These themes were refined and organized into a coherent narrative, providing insights into the experiences and challenges of FSDR implementation across South Africa’s provinces.

#### Quantitative analysis.

Descriptive statistics were applied to the extracted quantitative data, such as reporting frequencies or proportions of specific occurrences.Given the limited nature of the quantitative information, these results were integrated with the qualitative findings to provide a more holistic interpretation. Although primarily qualitative, the study employed an embedded mixed-methods design, wherein limited quantitative data were used to complement and enrich the qualitative findings. This integration allowed for a nuanced understanding of the document’s implementation and outcomes.

### Conceptual framework

The underlying conceptual framework for this study resulted in the data being analysed deductively according to the RE-AIM (Glascow, 2009) [22] framework, which has been shown to be an effective tool for evaluating the implementation of a policy from an implementation-sciences perspective [25]. This framework is illustrated in the figure below. Operational definitions were derived for this data analysis to ensure relevance to the FSDR implementation evaluation. The operational definitions are listed in Table 1.

**Table 1:** Operational definitions of the RE-AIM framework.

Re-AIM Component	Operational definition
Reach	The extent to which the FSDR implementation affected the end users (e.g., ground level clinicians, persons with disabilities etc). The end users of the FSDR were categorized as direct, indirect or intended.
Efficacy	The degree of effectiveness of the FSDR. This refers to how appropriate the FSDR was in achieving its main objective. i.e., to what extent has the FSDR increased access to rehabilitative health care for persons with disabilities
Adoption	This refers to the level of acceptance of the FSDR by it’s implementers, e.g.,; the number of collaborations formed as a result of the FSDR
Implementation	The degree to which the FSDR strategic plan and indicators were achieved.
Maintenance	The level at which the FSDR targets and objectives are sustainably implemented and continuously monitored and evaluated.

### Trustworthiness

Critics sometimes question trustworthiness in qualitative research; however, researchers have proposed strategies to ensure trustworthiness^23^. These strategies are credibility, confirmability and transaferability [23]. The credibility of this study was ensured through collaborative coding by the principal investigator and co-authors, whereby the final data analysis was reviewed to obtain a consensus on the final themes and subthemes. Additionally, an independent reviewer assessed the data analysis to confirm that the consensus obtained reflected the reality of the study. To ensure confirmability, this study ensured an in-depth description of the methodologies used and the use of the co-coders. Additionally, the final data analysis was reviewed by the NDOH to confirm the study findings. Moreover, transferability was ensured through the use of detailed descriptive methodologies used in this study.

### Ethical considerations

Ethical clearance was obtained from the Human Ethics Research Committee of the University of Witwatersrand (M220364), and permission was obtained from the National and Gauteng Department of Health through an online application on the National Health Research Database (GP_202202_059). The data in the reports were fully anonymised before the researcher accessed them and the ethics committee waived the requirement for informed consent.

## Findings

Eight out of nine provinces provided reports on the implementation of the FSDR in their contexts resulting in an 89% response rate. A few provinces highlighted that the Covid-19 pandemic negatively impacted the implementation of the FSDR, thus resulting in low levels of implementation. Some provinces provided more information than others, and some provinces provided values that others did not which is reflected in the graphics below. The findings highlight the uneven implementation of FSDR across different provinces in South Africa, with significant variations in access, training, and monitoring & evaluation. Most provinces received hard copies of the FSDR, but online access was limited, and training on its implementation was inconsistent, with staff shortages impacting service delivery. The absence of comprehensive monitoring and evaluation systems across most provinces and a lack of standardized training hindered effective implementation, with only one province actively reporting on M&E findings.

### Physical accessibility to the FSDR

**Fig 1:**
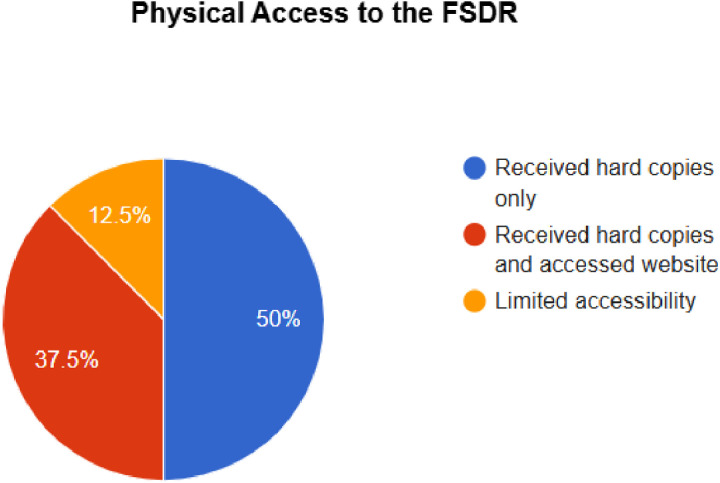
Percentage of provincial facilities with physical access to Functional Screening and Disability Rehabilitation (FSDR) services across eight provinces.

The physical accessibility to the FSDR document was determined by responses from provinces on whether or not they received hard copies of the FSDR and or accessed it online. This is seen in Fig 1 below.

### Training on FSDR implementation

**Fig 2:**
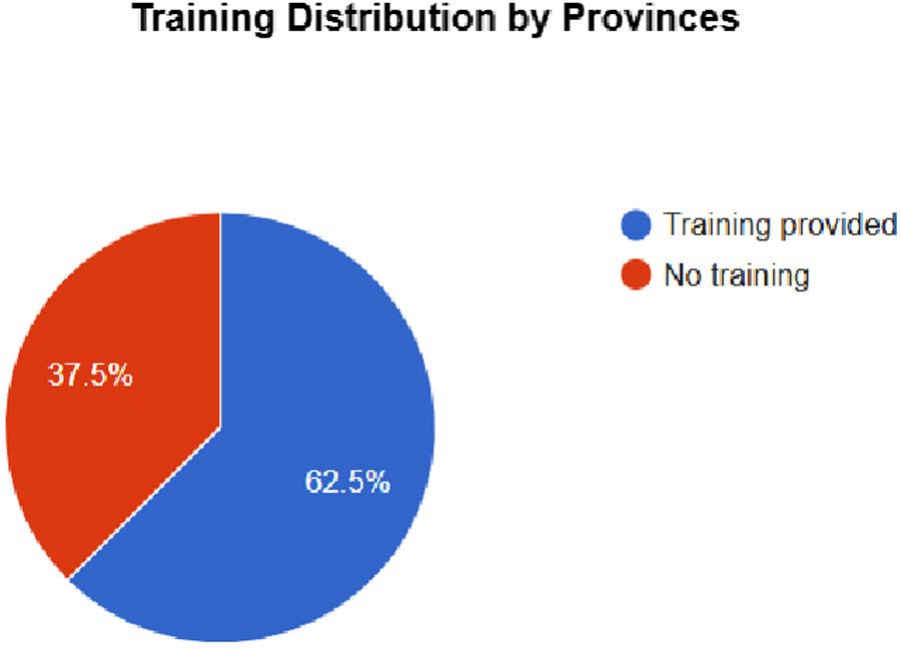
Provincial training status on FSDR implementation across eight provinces, indicating whether training was conducted or not.

**Fig 3:**
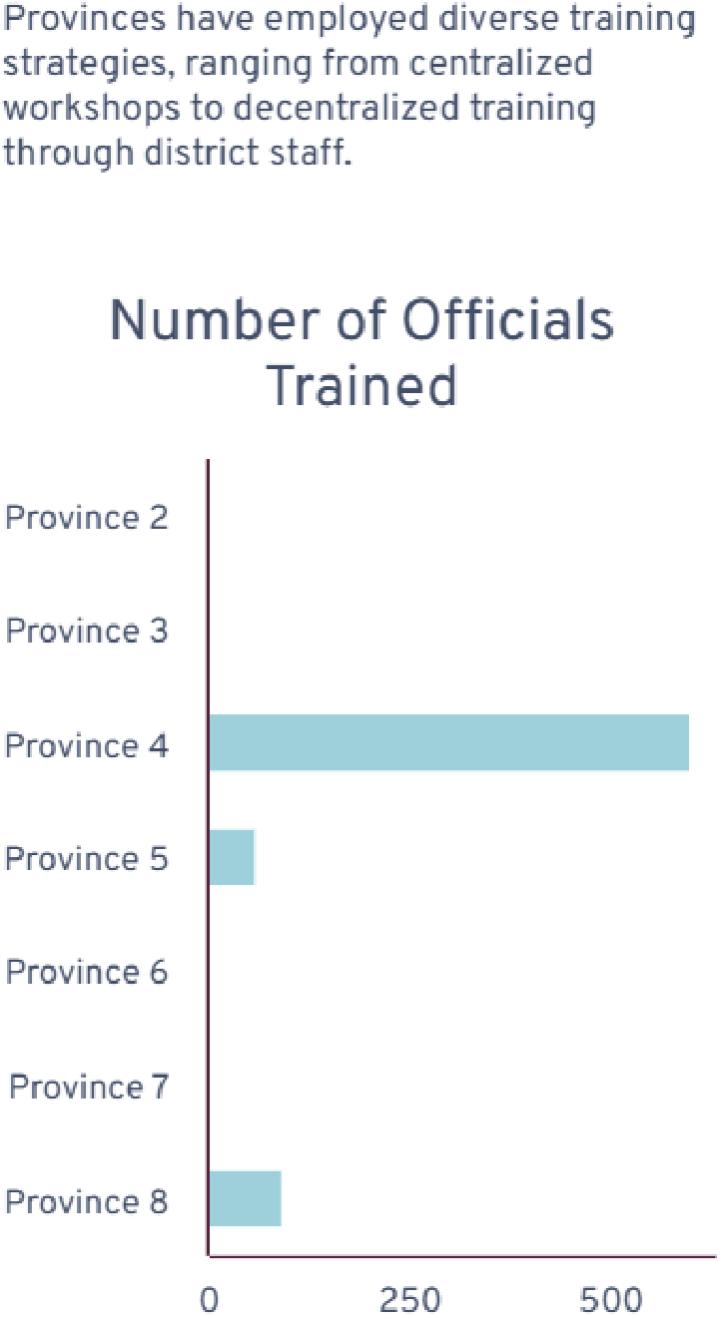
Number of rehabilitation professionals trained on FSDR implementation in three provinces that provided data.

The paper based report explored whether or not provinces conducted training on FSDR implementation. The responses to this are seen in Fig 2 below:

Of the 62.5% of provinces that conducted training on FSDR implementation, only 3 provinces provided quantified data on the number of rehabilitation professionals trained on the implementation of the FSDR. This is seen in Fig 3 below.

### M&E of FSDR implementation

**Fig 4:**
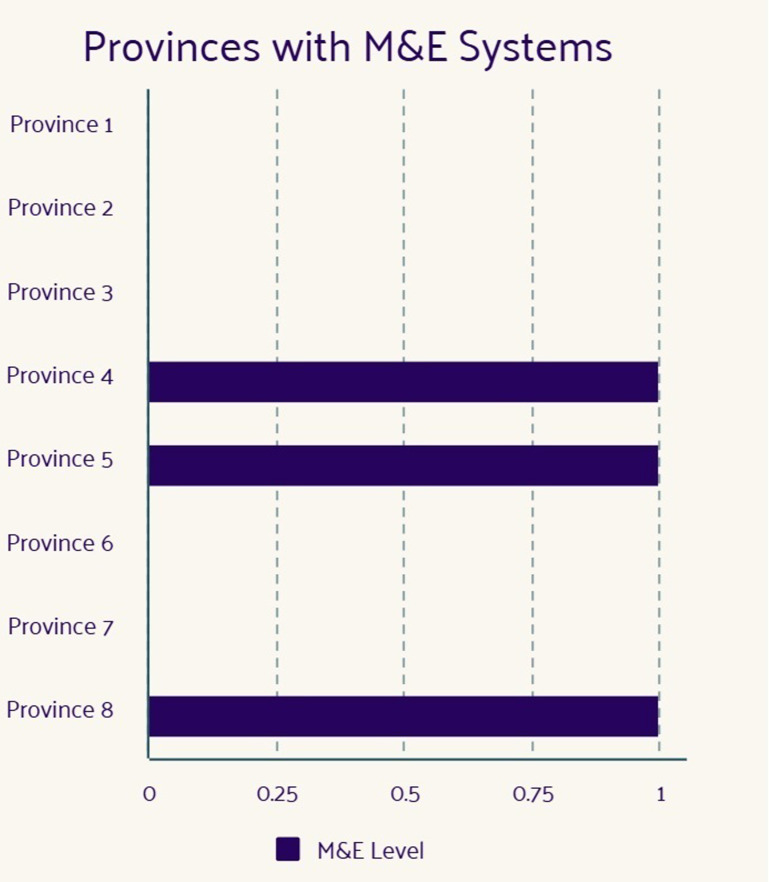
Monitoring and evaluation (M&E) reporting status for FSDR implementation across eight provinces.

The implementation report sought to understand if the FSDR implementation was monitored and evaluated in each province. Fig 4 below shows the number of provinces that conducted M&E of the FSDR implementation.

### Workforce for FSDR implementation

**Fig 5:**
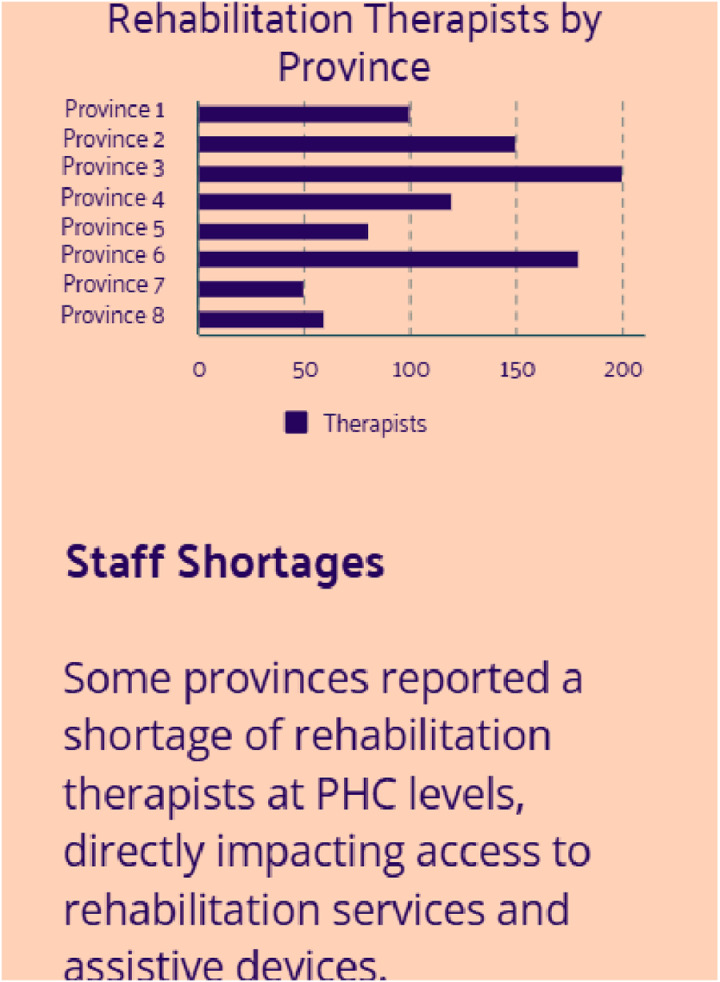
Availability of rehabilitation professionals to support FSDR implementation across eight provinces.

**Fig 6:**
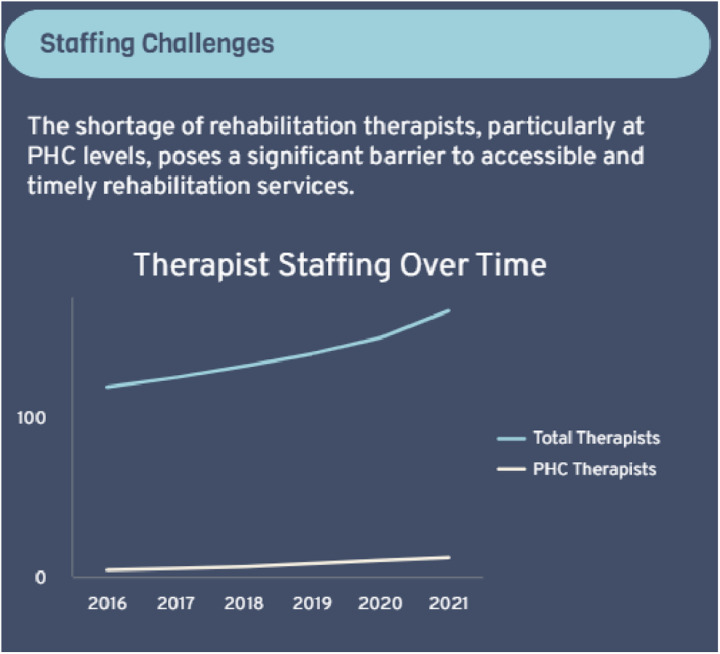
Proportional distribution of rehabilitation staff at Primary Health Care (PHC) levels relative to all available rehabilitation professionals.

Provinces reported on how many therapists were available to support the implementation of the FSDR. These values are seen in Fig 5 below:

In addition to provinces providing information on the number of rehabilitation professionals available to support FSDR implementation, provinces also provided information on the lack of staff at primary health care (PHC) levels which is primarily where the FSDR implementation was focused at. This is seen in Fig 6 below.

The second section of the FSDR report on implementation from each province focuses on the eight goals and the correlating objectives outlined in the FSDR. Responses to the realisation of these goals were categorised into main themes. An overview of the provincial reports using the RE-AIM framework is shown in Table 2.

**Table 2:** Overview of findings according to the Re-Aim Glasgow (2009) framework.

Framework component	Subtheme	Document quote
Reach	FSDR implementation training	*“Orientation for Therapeutic and Rehabilitation professionals to the FSDR was done with the involvement of NDoH Disability, Rehabilitation and Older Persons Manager in 2017” [* 1 *]*
	Lack of orientation	*“There were no orientation and support that took place.” [* 7 *]*
	Lack of handover	*“The province was orientated and supported to implement the Framework during 2015. There was however many changes in Management structures within the province. There was no carry over between these structures. “[* 2 *]*
Efficacy	Inclusive priority programmes	*“(Priority) Programmes were all orientated on the need to include disability and rehabilitation on their plans. But this process is done continuously by the districts.” [* 5 *]* *“KZN has achieved 50% of priority programmes that reflect disability and rehabilitation services in operational plans.” [* 6 *]*
	Accessibility	*“Annual reviews of* *accessibility standards are conducted, and* *adjustments/improvements as needed.” [* 3 *]* *“The Province participate(d) in scheduled clinical awareness workshops that are conducted by facilities and provide information on disability matters.” [* 5 *]*
	Establishment of referral pathways	*“The successful implementation of the existing referral pathway for cerebral palsy and other related cases.” [* 6 *]*
Adoption	Intersectoral collaboration	*“Mental Health services are inclusive of persons with disabilities and programmatic planning is specifically inclusive on persons with intellectual disabilities which is evidenced in activities such as the Right to Education intersectoral task team.” [* 3 *]* *“Rehabilitation services reflects in the EC Department of Health APP,ECDOH operational plan,MEC priorities,District health services plan and legal services (medico-legal strategy)plan. “[* 4 *]*
	Screening across levels of care	*“Developmental screening occurs at household, PHC clinic and hospital out-patient department.” [* 6 *]*
Implementation	Resource limitations	*“lack of rehabilitation coordinators at district level affected the rollout of FSDR training and provision of appropriate support at facility level.” [* 8 *]*
	Stratified implementation	*“In [Province 6], the disability and rehabilitation collaboration with different stakeholders including provincial, district and local government spheres as well as disability sector and civil society through the provincial disability forum which is housed in the office of the premier.”[* 6 *]*
Maintenance	Disability-related data aggregation	*“lack of disaggregation of disability-related data in departmental monitoring tools.” [* 8 *]*
	Lack of M&E tools	*“no M&E tool was received from NDoH but as [Province 1], a Rehab Strategy (although unsigned) which encompassed the FSDR goals was developed (to monitor) implementation.” [* 1 *]*

### Reach

A major factor of reach relates to the level of training which was conducted throughout each province on the FSDR. Some provinces provided numerical values for the number of individuals trained, whereas others provided insight into who the trainers were and which rehabilitation professionals were trained. An example of this is seen in the Eastern Cape, with 600 officials being trained, followed by the Northwest province, with 93 officials being trained, and Mpumalanga, with 60 officials being trained. The other provinces that did not provide numerical values still provided insight into the fact that some training was conducted by the NDOH on provincial rehabilitation managers. In turn, provincial rehabilitation managers trained profession-specific district rehabilitation coordinators, including physiotherapy (PT), Occupational Therapy (OT), speech therapy (ST), Audiology, Medical Orthotics, prosthetics, and podiatry. Another noteworthy point was the inclusion of community service physiotherapists during their annual induction sessions. Table two above showcases the experiences of rehabilitation providers in the FSDR implementation with one province stating that “The province was oriented and supported the implementation of the Framework in 2015. There was however many changes in Management structures within the province. There was no carry over between these structures.” This shows that despite efforts to train end users, the lack of carryover internally affected implementation outcomes.

Additionally, FSDR’s reach is defined by the level of physical accessibility to the document itself. This refers to the extent to which an actual document is reached. The majority of the provinces stated that they received hard copies of the FSDR from the NDOH, whereas others accessed the FSDR electronically through the NDOH website. Most of the provinces utilised electronic copies of the FSDR, as it was more accessible.

Moreover, the reach of the FSDR spoke to the number of intended, direct, and indirect individuals affected by the FSDR implementation. The FSDR was intended to target rehabilitation managers and therapists, and this was achieved directly. The FSDR also indirectly affected the improvement in rehabilitation access for communities that may have been isolated prior to FSDR implementation. The extent to which indirect and unintended individuals were affected was unclear from the paper-based evaluation, as further investigation is required.

### Efficacy

The efficacy of the FSDR refers to the extent to which the FSDR increases access to rehabilitative healthcare for persons with disabilities. More ground-level investigations are required to understand the level of accessibility. The provinces described that their facilities were already physically accessible and that they reviewed and aligned them to national accessibility standards. Some provinces conducted accessibility audits, but no clear outcome was provided, as these are still being conducted. In addition, the provinces have developed guidelines to improve the issue rates of assistive devices. This was seen by one province that stated that they have “…*achieved 50% of priority programmes that reflect disability and rehabilitation services in operational plans.”* This was a positive result of the FSDR implementation in the province.

### Adoption

This refers to the level of acceptance of the FSDR by its implementers, for example, the number of collaborations formed as a result of the FSDR. This primarily speaks to Goal 3 which seeks to foster intersectoral collaboration. The majority of the provinces developed intersectoral and interprofessional relationships to improve access to rehabilitation for persons with disabilities. A clear example of this is the numerous partnerships with local communities and non-profit organisations which facilitated accessibility. Additionally, an example of this is in the Northwest province, where the provincial department of health was working with the Department of Women, Youth, and Persons with Disabilities. Mpumalanga also established relationships with the Office on the Status of Disabled People. In some provinces, the hosting of provincial disability forums has facilitated collaboration.

### Implementation

The implementation of the FSDR spoke to its effectiveness in achieving its targets, objectives, and indicators. The framework defined in the FSDR and utilised in the actual paper-based evaluation template described eight main goals with correlating objectives. These goals sought to integrate rehabilitation into priority health programmes, develop effective rehabilitation referral pathways, foster inter-sectoral collaboration, implement accessibility standards, increase the awareness levels of disability, improve the M & E of disability services, improve human resources for rehabilitation services, and improve access to assistive devices.

The exact extent to which these were achieved varied between provinces owing to the different ways in which the provinces responded. One province, Limpopo, responded with quantitative values regarding each goal and its objectives by defining the responses based on the number of facilities and individuals targeted. In most cases, the realisation of all goals was halted due to the Covid-19 Pandemic.

### Maintenance

The level at which the FSDR targets and objectives are being sustainably implemented and continuously monitored and evaluated. Most provinces did not monitor or evaluate the FSDR. Some provinces stated that they did not have a tool for M&E, whereas others did not state why they had not conducted M & E. In some instances, the provinces quantified their M & E by providing amounts for their wheelchair issue rate of 78%.

The above section provides an overview of FSDR implementation in relation to the RE-AIM framework. The next section discusses the barriers and facilitators experienced by the provinces during the implementation of FSDR.

## Discussion

This study aimed to review the reports on the implementation provided by each province. To our knowledge, this is the only study to review these reports and provide a bird’s eye overview of FSDR implementation across different provinces. The National Department of Health reviewed eight provincial reports [24].

The success of any health policy depends on how well equipped implementers are at ground level. From the results, it was difficult to quantify the level of reach to ascertain how far the FSDR went and whom it actually reached. There was a fair degree of physical accessibility to the FSDR among management and coordinators, but it is unknown how many clinicians at the ground level had access to implement the FSDR. A study [24] argued that it is highly necessary for ground-level end users (in this case, clinicians and managers), to be involved in the implementation of a policy from the inception of implementation. In the case of FSDR, a top-down approach was used which may have hindered further implementation. In contrast, another study [25] explained that a top-down approach may not necessarily be a negative factor in disability policy implementation, provided that it allows end users to openly engage in their experience of implementation.

### Efficacy

The FSDR sought to increase accessibility for people with disabilities by increasing the physical and geographical accessibility of the services provided. Interestingly, provinces reported that their facilities were already accessible, and all that was required for FSDR implementation was the alignment of services to the policy. It may arguable as to how these provinces perceived “accessible” considering the heterogeneity of rehabilitation facilities across South Africa. A study [26] justifies that policy implementation is not only reliant on the efficacy of the policy and its content but also on the self-efficacy of the implementer. This was also explored by an author [27], who showcased a participatory action-based project which highlights how policy implementers can be powerful in influencing policy and progress of efficacy of implementation when they are adequately equipped for implementation and willing to implement. However, we acknowledge the need for further quantified evidence to establish whether the FSDR achieved optimal efficacy through its implementation.

### Adoption

The adoption of any policy requires a certain degree of acceptance of the policy content among the implementers [27,28]. A major output for the adoption of the FSDR was the development of intersectoral collaboration which some provinces reported to be able to achieve. Again, the extent to which they achieved this is unknown. Authors [29] describe how adoption of policy improves when there is implementation support. However, it should be noted that this study was conducted in a European context and may not accommodate contextual differences in South Africa; therefore, the findings cannot be generalized.

### Implementation

Implementation science is emerging in the field of rehabilitation, and the ability of policies to be adequately implemented according to the policy contents requires further exploration [30]. While the responses were fairly general and not quantified, differences were noted in the ways in which provinces implemented the FSDR. Examples of these differences are seen where one province implemented the FSDR by developing and implementing additional internal policies, such as the Gauteng Disability Policy and Roadshow [30]. Another province explained their implementation by conducting accessibility audits to ensure that rehabilitation facilities were physically accessible to persons with disabilities. There may be numerous reasons for the heterogeneity of implementation across provinces, including the understanding of accessibility in each province coupled with each province operationalizing the FSDR according to their contexts. This highlights the need for a disability policy that can adapt to different environments. Moreover, these differences showcase flaws in the training of end users in the policy implementation process, as standardized implementation measures may have resulted in similar implementation outcomes across the country.

These differences speak to the findings of a study [31], that compared the findings of policy implementation in South Africa and Kenya and found that the biggest factor affecting implementation was the differences in governance of health services at different levels, as is the case of rehabilitation management at the national, provincial, and district levels during the FSDR implementation. As such, we propose adequate, standardized training on implementation within each of the rehabilitation governing structures of each province, coupled with standardized monitoring and evaluation tools to assess the same outcomes.

### Maintenance

Monitoring and evaluating policy implementation efforts are among the most crucial aspects for ensuring successful policy implementation [32]. In our study, provinces reported that a lack of available M&E indicators resulted in no M&E activities being conducted in some provinces. The authors [32] explain that this is often an oversight in many policies due to the absence of monitoring tools to continuously evaluate progress on implementation. This finding also shows a flaw in the development of the FSDR: no M&E tool was developed at the development stage alongside the formulation of the policy content, which affected the implementation of the FSDR. This finding is supported by other authors [33,34,35], who emphasized the need for monitoring tools to be developed at the early stages of policy development, highlighting the need for these tools and indicators to be based on a participatory approach to ensure that end users are included in the tool design.

### Strengths

This study is the first to evaluate the implementation of the FSDR based on the provincial implementation report, providing a broad overview of the FSDR implementation. The inclusion of end users and NDOH in the study design provided a participatory research approach to ensure that the study understood the research question and matched the research methods accordingly.

### Study limitations

The data used in this study were retrospective data collected by the National Department of Health which may pose a risk of low-quality data and responses. Additionally, the reports reviewed only account for the experience of implementation at provincial management levels and not at ground level which may not be a true reflection of FSDR implementation among end users.

### Recommendations

According to the study findings, further investigation is required at the ground level to assess the implementation of the FSDR amount by end users to understand their experiences and perceptions. Further efforts should be made to quantify the implementation of the FSDR to inform future policies. Moreover, the barriers identified in this study, such as resource limitations, should be addressed through capacity-building initiatives.

## Conclusion

The FSDR has not achieved its full level of implementation owing to multiple barriers, such as poor implementation training, lack of resources, and human capacity. Facilitators, such as intersectoral collaboration, should be leveraged to improve disability policies to realise equitable access to rehabilitation services for people with disabilities.
